# Aortic Dissection Presenting as Acute Pancreatitis: Suspecting the Unexpected

**DOI:** 10.1155/2018/4791610

**Published:** 2018-01-30

**Authors:** Adam Hafeez, Dillon Karmo, Adrian Mercado-Alamo, Alexandra Halalau

**Affiliations:** ^1^Internal Medicine Department, Beaumont Hospital, Royal Oak, MI, USA; ^2^Oakland University William Beaumont School of Medicine, Rochester, MI, USA; ^3^General Internal Medicine Division, Beaumont Hospital, Royal Oak, MI, USA

## Abstract

Aortic dissection is a life-threatening condition in which the inner layer of the aorta tears. Blood surges through the tear, causing the inner and middle layers of the aorta to separate (dissect). It is considered a medical emergency. We report a case of a healthy 56-year-old male who presented to the emergency room with sudden onset of epigastric pain radiating to his back. His blood pressure was 167/91 mmHg, equal in both arms. His lipase was elevated at 1258 U/L, and he was clinically diagnosed with acute pancreatitis (AP). He denied any alcohol consumption, had no evidence for gallstones, and had normal triglyceride level. Two days later, he endorsed new suprapubic tenderness radiating to his scrotum, along with worsening epigastric pain. A MRCP demonstrated evidence of an aortic dissection (AD). CT angiography demonstrated a Stanford type B AD extending into the proximal common iliac arteries. His aortic dissection was managed medically with rapid blood pressure control. The patient had excellent recovery and was discharged home without any surgical intervention.

## 1. Introduction

Aortic dissection remains the deadliest manifestation of acute aortic syndromes with mortality as high as 1% per hour for acute type A aortic dissections [1]. The classical clinical presentation of an aortic dissection is of a male in his 60s who presents to the emergency room with sudden onset tearing or ripping chest pain. Hypertension, noted on vitals and with physical exam maneuvers such as blood pressure discrepancies in the arms, can help make the diagnosis. However, more importantly, the presentation can vary, and the classical symptoms may be absent or inconsistent [2]. This can paint a diagnostic dilemma for the clinician.

Acute pancreatitis has been increasing in incidence, with estimates of an increase in 20% in admissions over the last 10 years in the United States [3]. Acute pancreatitis also presents with severe epigastric pain which often radiates to the back. Diagnosis can be made with two of the three following features: (1) abdominal pain, (2) elevated serum lipase or amylase at least three times the upper limit of normal, and (3) characteristic findings of acute pancreatitis on contrast-enhanced computed tomography (CT), magnetic resonance imaging (MRI), or even ultrasound [4]. Therefore, the diagnosis can be made clinically without imaging.

The presentation of acute pancreatitis in the setting of aortic dissection poses a diagnostic challenge, as some of the cardinal symptoms of aortic dissection, such as hypertension and pain, overlap with acute pancreatitis. Acute pancreatitis is a clinical diagnosis, but without an easily identifiable cause, such as gallstones, alcohol abuse, or hypertriglyceridemia, further imaging may be warranted if the patient does not clinically improve. Due to its lethal complications, physicians should be aware and should have a high index of suspicion for aortic dissection when evaluating a patient with presumed acute pancreatitis.

Acute pancreatitis presenting as acute aortic dissection is a rare entity with less than ten well-documented cases reported so far. It is hypothesized that the pancreas can be susceptible to hypoperfusion as seen in cardiopulmonary bypass surgery [3]. We present a case misdiagnosed as only acute pancreatitis based on clinical symptoms and later correctly diagnosed as an aortic dissection when symptoms did not improve.

## 2. Case Presentation

**Figure 1:**
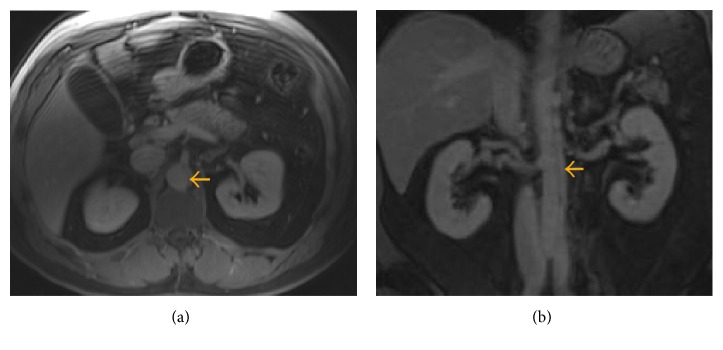
Magnetic resonance cholangiopancreatography (MRCP). (a) Dissection can be seen extending to the origin of the iliac arteries. (b) MRCP demonstrating acute aortic dissection with narrowing of the true lumen (yellow arrow). Of note, there is no peripancreatic inflammatory changes or fluid collections noted.

**Figure 2:**
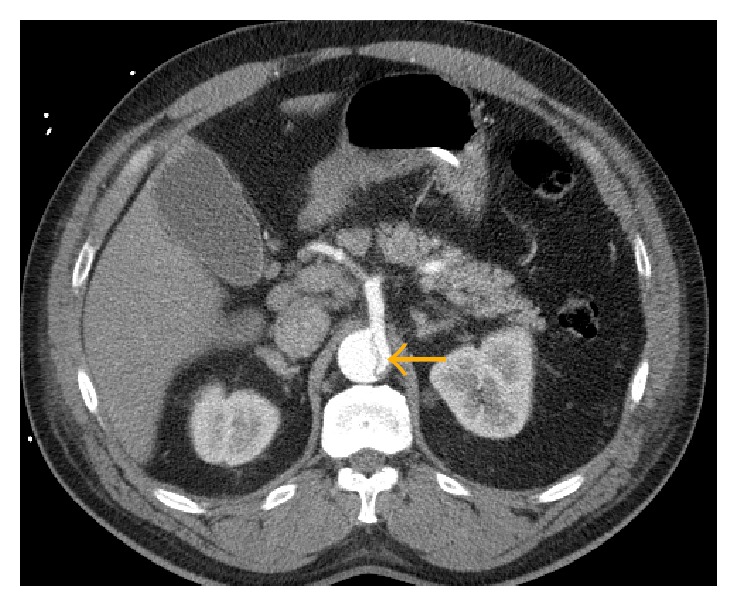
CT angiography showing acute type B aortic dissection with severe narrowing of the true lumen (yellow arrow).

**Figure 3:**
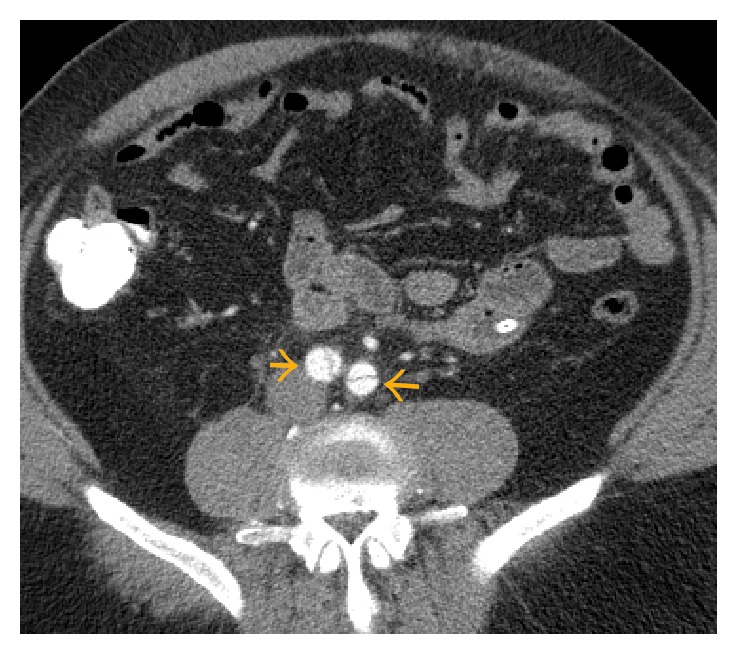
CT angiography showing acute type B aortic dissection extending into the proximal common iliac arteries (yellow arrows). This parallels with the patient's clinical complaints of scrotal pain and dysuria.

A 56-year-old male with no significant past medical history presented to the emergency room with the chief complaint of sudden onset of substernal chest pain radiating to his back. His family history was noncontributory. He denied tobacco, alcohol, or illicit drug use. The patient worked as a traveling musician. He denied any recent trauma. Initial vitals revealed hypertension (167/91 mmHg). Physical exam was significant for reproducible midepigastric pain, without guarding or rebound. Chest and abdominal radiographs were unremarkable. The patient had a mild leukocytosis (10.9 bil/L), elevated lipase (1258 U/L), elevated amylase (163 U/L), normal triglycerides, and negative autoimmune workup (Table 1). His EKG showed normal sinus rhythm with a rate of 80 beats/minute, normal axis, normal interval durations, and no signs of acute or chronic ischemia or left ventricular (LV) hypertrophy. Abdominal US was negative for cholelithiasis. He had no recent procedures performed and was on no medications prior to presentation. He was treated with IV fluids and bowel rest for presumed acute pancreatitis diagnosed clinically because of elevated lipase and epigastric pain. An abdominal CT scan to assess the severity of pancreatitis was not indicated to be performed as the patient's clinical symptoms were stable. Throughout his admission, his pain never fully resolved. On day 5, he developed a marked increase in blood pressure (194/112 mmHg). He was diaphoretic on exam with worsening of his epigastric pain. He also endorsed new suprapubic pain radiating to his scrotum along with dysuria and increased urinary frequency. Because of the acute worsening of the patient's condition, a MRCP was ordered to examine the pancreas and the hepatobiliary tree. Incidentally, the MRCP demonstrated evidence of a large aortic dissection (Figure 1). CT angiography demonstrated a Stanford type B AD (Figure 2) extending into the proximal common iliac arteries (Figure 3) that explained his new suprapubic and scrotum pain. There was no radiographic evidence of peripancreatic inflammatory changes or fluid collections.

The patient's pancreatitis was treated with bowel rest, IV fluids, and pain medication. The patient's aortic dissection was treated with IV labetalol and nitroprusside in the ICU with rapid lowering of his blood pressure. No surgical intervention was performed.

## 3. Discussion

Aortic dissection remains a devastating vascular catastrophe with a high mortality rate if not diagnosed and managed promptly. However, its signs and symptoms are unpredictable. As the dissection progresses, other organs can be affected and can obscure the inciting diagnosis. Pancreatitis remains a rare complication of aortic dissection, and the association between the two has yet to be elucidated. A retrospective study by Li et al. examining the forensic characteristics of patients who suffered sudden unexpected deaths due to an aortic dissection showed that 83.9% of the patients were misdiagnosed and the dissection was not recognized. Of these, only one case was diagnosed as acute pancreatitis, which highlights the rarity of this presentation [5]. To our knowledge, there are only ten well-documented cases reported worldwide. Recently, Pham and Nable described a case of a woman with an exacerbation of several months of abdominal pain that was clinically diagnosed with pancreatitis based on epigastric pain as well as elevated lipase (723 U/L). Unlike our case, the woman had a prior history of aortic aneurysmal repair. An abdominal CT was pursued to look for any complications of pancreatitis. She was found to have an incidental Stanford type B aortic dissection [6].

The International Registry of Acute Aortic Dissection (IRAD) database recognizes malperfusion in 21% of the patients with Stanford type B aortic dissection. However, acute pancreatitis is not described as an ischemic complication of acute aortic dissection in the IRAD [7]. Common risk factors for acute pancreatitis are gallbladder disease, chronic alcohol consumption, and recent procedures. Rarely, hypercalcemia, hyperlipidemia, autoimmune disorders, or medications can induce pancreatitis [3]. A limited number of case studies reported an association of acute aortic dissection with acute pancreatitis. Masaki reports a case of a middle-aged man diagnosed first with Stanford type B aortic dissection with the true lumen supplying the celiac trunk and splenic artery. Due to thrombosis of the false lumen, the true lumen became narrow. The patient developed upper abdominal pain as well as an elevation in pancreatic amylase. A CT showed peripancreatic fluid [8]. These findings were consistent with acute pancreatitis. As the acute pancreatitis developed hours after aortic dissection, it was thought that possible ischemia-reperfusion injury initiated the acute pancreatitis. A similar case was described by Zheng et al; however, the patient was found to have a type A aortic dissection when a CT scan was performed to confirm the diagnosis after management of acute pancreatitis had already begun [9].

Ischemic injury to the pancreas occurring in clinical situations such as shock or major cardiac surgery is rare. For example, ischemic injury to the pancreas following cardiopulmonary bypass surgery has been reported to occur with an incidence of less than 1% [10]. Similarly, resection or repair of an aortic aneurysm has been shown to induce pancreatitis [11]. Aortic dissection can, like cardiopulmonary bypass surgery or manipulation of the aorta, produce a hypoperfused state leading to ischemic injury to the pancreas. However, the injury seen in the pancreas may not be as severe as other organs, such as the kidney or liver. It is hypothesized that this can be due to rich collateral blood supply to the pancreas [12]. Notably, the pancreatic head receives blood supply from the superior mesenteric artery. The body and tail, on the other hand, can receive blood not only from branches of the splenic artery but also from branches originating from the celiac or superior mesenteric arteries as well as the splenic hilum itself [12]. This may explain why the acute pancreatitis caused by hypoperfusion may remain mild and not produce radiographic evidence of pancreatitis, as seen in our patient. Additionally, an acute aortic dissection is a dynamic process, and it is possible that transient malperfusion may take place as the dissection progresses. Therefore, the lipase may reflect ischemic injury that has resolved.

Due to the arterial collaterals supplying the pancreas, it is likely that hypoperfusion may not be the only factor at play. Another hypothesis comes from a case report of a patient that suffered severe necrotic pancreatitis caused by a type B aortic dissection [13]. It was hypothesized that cholesterol emboli from the dissecting aorta were the main cause of the pancreatic necrosis [13]. In our case, there was no such evidence for any embolic phenomena.

As noted, the kidney, heart, brain, and liver are commonly affected by shock. The pancreas is not regularly believed to be affected, although pathophysiologically can suffer ischemia like any other organ. Warshaw and O'Hara et al. retrospectively studied autopsies of patients who died due to shock after repair of abdominal aortic aneurysms or open heart surgery. He found a 9% incidence of pancreatic injury in this patient group. More interestingly, there was a strong positive correlation with presence of renal ischemic injury, with 50% concomitant pancreatic injury in the setting of acute tubular necrosis (*p* < 0.001) [14]. The author noted that it was ischemia, not operative trauma, which led to pancreatic injury. More so, all the patients with shock that were followed prospectively had elevated lipase levels but no signs or symptoms of pancreatitis. This is similar to our case report in the sense that the patient had elevated lipase and aortic dissection-related pain but no evidence of acute pancreatitis on imaging.

As the precise etiology of pancreatitis following aortic dissection continues to be elucidated, what remains is the diagnostic challenge of efficiently differentiating between the clinical manifestations of aortic dissection and other etiologies of acute abdominal pain such as acute cardiac ischemia, pancreatitis, or intestinal obstruction. Imaging remains at the heart of this differentiation, but this may not always be cost-effective, efficient, and noninvasive. Most patients with severe hypoperfusion will be having renal ischemia, and adding contrast agents for imaging may not be ideal. Wen et al. researched the role of matrix metalloproteinases (MMPs), proteases that have been indicated in aortic aneurysms to correlate with aortic-wall proteolysis, as a diagnostic tool for detection of aortic dissection. MMPs also have been shown to be elevated in patients with pancreatitis. They prospectively studied 20 patients each with aortic dissection and acute pancreatitis within 1 hour of symptom onset in the emergency room and found that MMPs were higher in patients with acute AD as compared to acute pancreatitis (*p* < 0.05) [15]. It is possible that, in the future, MMPs to be used as a marker to help differentiate between the two causes of severe epigastric pain. Although more research into optimal markers and imaging needs to be done, the burden rests on the clinician's shoulders to keep a wide differential diagnosis. If an etiology is not identified, further imaging must be pursued sooner rather than later to rule out rarer etiologies for pancreatitis and to also assess for other possible etiologies in the same anatomical region, that is, aortic dissection.

## Figures and Tables

**Table 1:** Key laboratory investigations on admission.

Laboratory	Value	Normal	Laboratory	Value	Normal
Hemoglobin	14.4	13.6–17.6 g/dL	AST	59	10–37 U/L
Potassium	3.6	3.5–5.3 mmol/L	ALT	111	9–47 U/L
BNP	10	0–100 pg/mL	Cholesterol	220	70–199 mg/dL
BUN	16	8–23 mg/dL	Triglyceride	111	40–139 mg/dL
Creatinine	1.16	0.64–1.27 mg/dL	Troponin	0.03	0.00–0.05 ng/mL
Amylase	163	20–104 U/L	ANCA	<1 : 20	<1 : 20
Rheumatoid factor	Negative	Negative	ANA screen	Negative	Negative

## References

[1] Coady M. A., Rizzo J. A., Goldstein L. J., Elefteriades J. A. (1999). Natural history, pathogenesis, and etiology of thoracic aortic aneurysms and dissections. *Cardiology Clinics*.

[2] Criado F. J. (2011). Aortic dissection: a 250-year perspective. *Texas Heart Institute Journal*.

[3] Forsmark C. E., Swaroop Vege S., Wilcox C. M. (2016). Acute pancreatitis. *New England Journal of Medicine*.

[4] Banks P. A., Bollen T. L., Dervenis C. (2013). Classification of acute pancreatitis—2012: revision of the Atlanta classification and definitions by international consensus. *Gut*.

[5] Li Y., Li L., Mu H.-S., Fan S.-L., He F.-G., Wang Z.-Y. (2015). Aortic dissection and sudden unexpected deaths: a retrospective study of 31 forensic autopsy cases. *Journal of Forensic Sciences*.

[6] Pham T. V., Nable J. V. (2013). Aortic dissection presenting with pancreatitis. *The American Journal of Emergency Medicine*.

[7] Suzuki T., Mehta R. H., Ince H. (2003). Clinical profiles and outcomes of acute type B aortic dissection in the current era: lessons from the international registry of aortic dissection (IRAD). *Circulation*.

[8] Hamamoto M. (2012). Acute ischemic pancreatitis associated with acute Type B aortic dissection: a case report. *Annals of Vascular Diseases*.

[9] Ziyu Z., Liu J., Huang Y. (2014). Acute pancreatitis induced by acute Type A aortic dissection: a case report. *Journal of Cardiovascular Disease Research*.

[10] Lefor A. T., Vuocolo P., Parker F. B., Sillin L. F. (1992). Pancreatic complications following cardiopulmonary bypass. Factors influencing mortality. *Archives of Surgery*.

[11] Sriussadaporn S. (1999). Acute pancreatitis following resection of juxtarenal abdominal aortic aneurysm. *Journal of the Medical Association of Thailand*.

[12] Okahara M., Mori H., Kiyosue H., Yamada Y., Sagara Y., Matsumoto S. (2010). Arterial supply to the pancreas; variations and cross-sectional anatomy. *Abdominal Imaging*.

[13] Umeda I., Hayashi T., Ishiwatari H. (2011). A case of severe acute pancreatitis and ischemic gastropathy caused by acute aortic dissection. *Nihon Shokakibyo Gakkai Zasshi*.

[14] Warshaw A. L., O’Hara P. J. (1978). Susceptibility of the pancreas to ischemic injury in shock. *Annals of Surgery*.

[15] Wen T., Liu L., Xiong G. (2009). Matrix metalloproteinase levels in acute aortic dissection, acute pancreatitis and other abdominal pain. *Emergency Medicine Journal*.

